# Supplementary material for: Regulatory basis for reproductive flexibility in a meningitis-causing fungal pathogen
Source: Nat Commun. 2022 Dec 24;13:7938. doi: 10.1038/s41467-022-35549-y (PMC9790007; doi:10.1038/s41467-022-35549-y)
Supplement: Supplementary file 1 — Supplementary Informantion [file 41467_2022_35549_MOESM1_ESM.pdf]

Regulatory basis for reproductive flexibility in a meningitis-causing fungal pathogen

Pengjie Hu[1], Hao Ding[1,2], Huimin Liu[1,3], Yulin Yang[1,2], Lei Chen[1,2], Guang-Jun He[1], Weixin Ke[1,2], Ping Zhu[4], Xiuyun Tian[1], Yan Peng[4], Zhenghao Shen[1,2], Xiaoxia Yao[1,2], Changyu Tao[5], Ence Yang[5], Guojian Liao[4], Xiao Liu[1,2], Linqi Wang[1,2,3]*

[1]State Key Laboratory of Mycology, Institute of Microbiology, Chinese Academy of Sciences, Beijing 100101, China. [2]University of Chinese Academy of Sciences, Beijing 100049, China. [3]University of Science and Technology of China (USTC), Hefei 230026, China. [4]The Medical Research Institute, College of Pharmaceutical Sciences, Southwest University, Chongqing 400715, China. [5]Department of Microbiology, School of Basic Medical Sciences, Peking University Health Science Center, Beijing 100191, China. 32

These authors contributed equally: Pengjie Hu, Hao Ding, Huimin Liu.
*E-mail: wanglq@im.ac.cn

Supplementary Figures

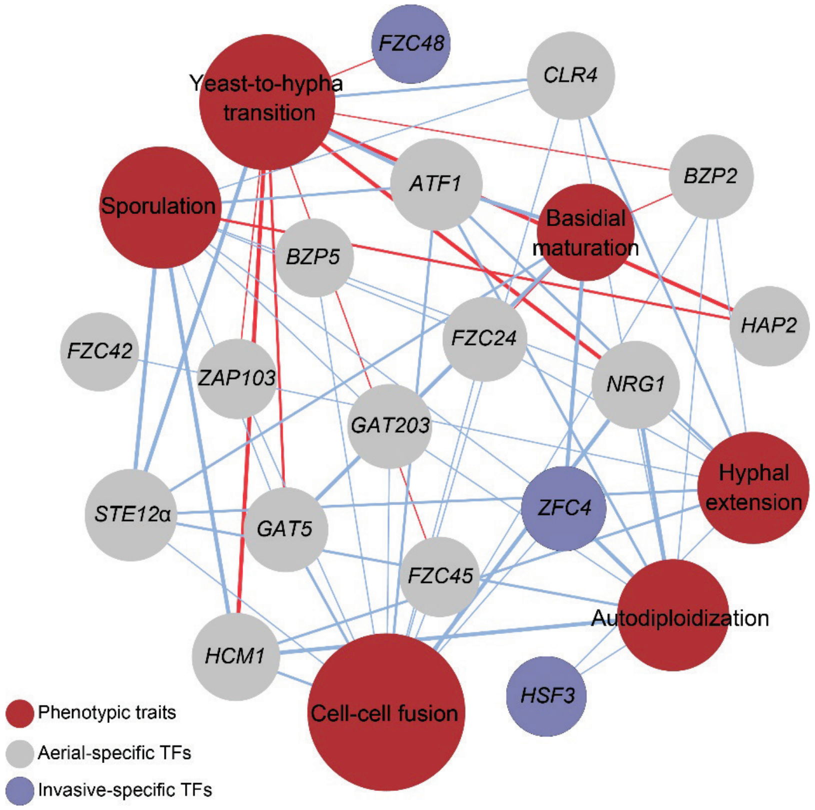

Supplementary Figure 1. The bipartite network links spatial TFs to the six phenotypic traits. Red nodes indicate the six phenotypic traits, and other nodes represent TFs. Nodes are sized according with their respective degrees of connectivity. The edges represent the phenotype strengths (strong, intermediate and weak) regulated by TFs, and red and blue edges indicate positive and negative regulation, respectively.

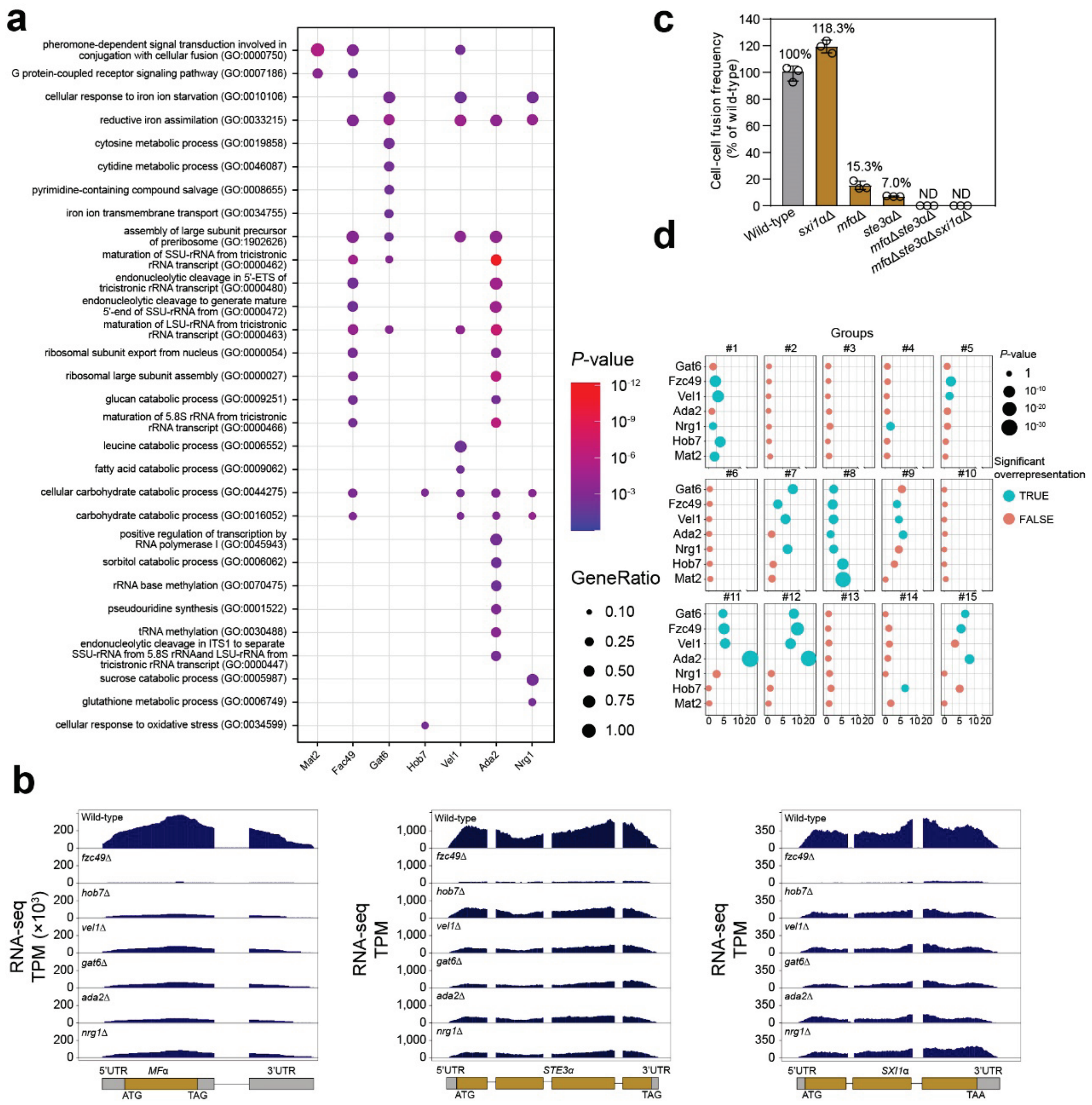

Supplementary Figure 2. Syngamy TFs act in concert in transcriptional induction a set of genes, including the sex-determination components.

a. Gene ontology (GO) biological process enrichment analysis of genes regulated by the seven syngamy TFs. Enriched gene ontology (GO) terms are shown with hypergeometric *P*-value and gene ratio.

b. Genome browser images depict relative transcript levels based on RNA-seq reads. Enriched RNA-seq signals visualized by Integrated Genome Browser are representative of two independent experiments. TPM, Transcripts per kilobase of exon model per million mapped reads. UTR, untranslated region.

c. Cell-cell fusion frequency of different mutants compared to wild-type. A unilateral cell-cell fusion assay was carried out between a mutant strain and a wild-type mating partner. Data are presented as the mean ± SD of three independent experiments.

d. Genes in group 8 are significantly enriched in the targets of seven TFs. x-axis displays the odds ratio of the enrichment of each TF regulons relative to genes in group 8 based on Fisher's exact test, and the y-axis represents different TFs. The blue circle indicates significant overrepresentation (one-sided Fisher's exact test, $P < 0.05$). The sizes of the circles correspond to the *P*-values from Fisher's exact test.

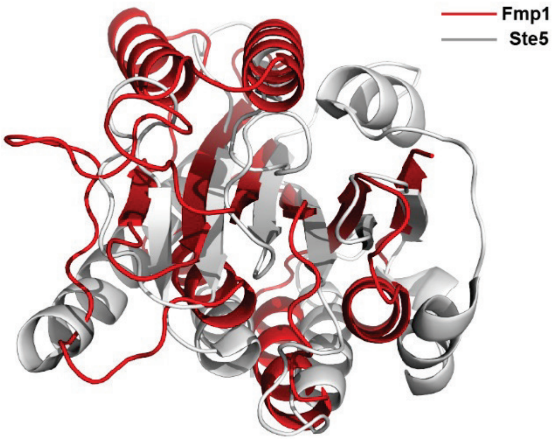

Supplementary Figure 3. AlphaFold2-generated vWA domain of Fmp1 (in red) superimposed on vWA domain of *Saccharomyces cerevisiae* Ste5 (in grey) with PDB ID: 4F2H.

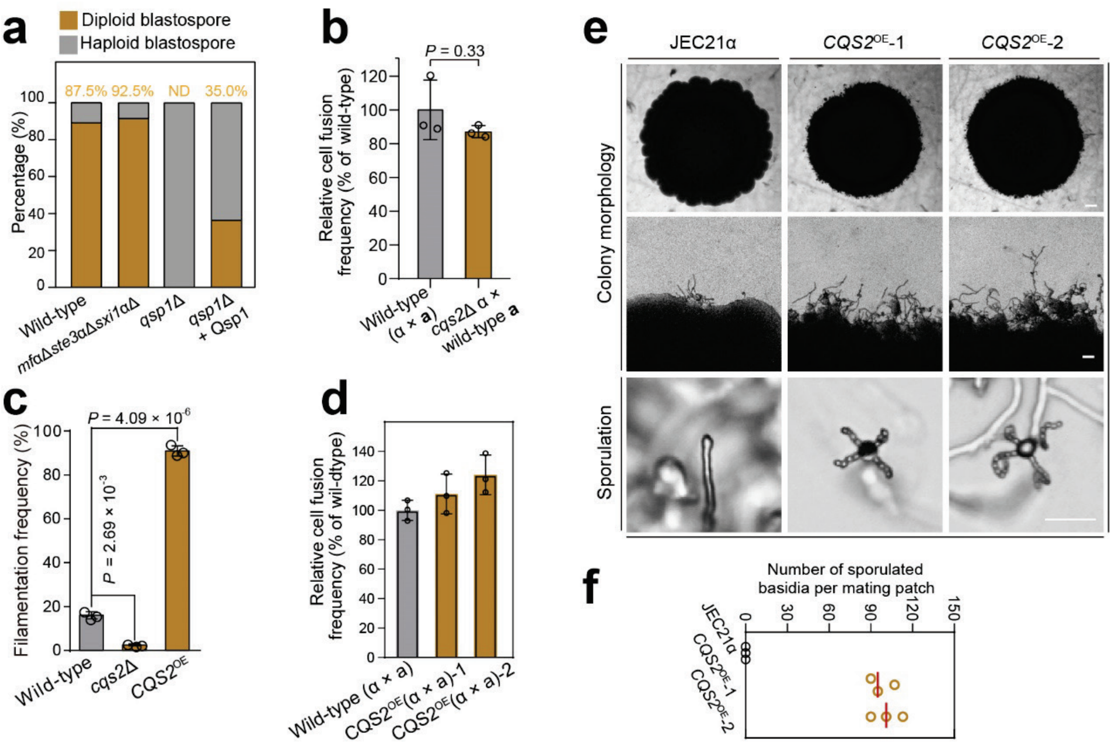

Supplementary Figure 4. **Cqs2 is dispensable for bisexual syngamy, but important for coordinating unisexual differentiation events.**

a. FACS-based ploidy estimation of blastospores dissected from unisexual hyphae of different strains. For each strain, 40 blastospores dissected from different unisexual hyphae were tested for ploidy level. ND, not detected.

b. Unilateral *cqs2* mutant cell fusion frequency compared to the wild-type strain. The data are presented as the mean ± SD from three independent experiments, two-tailed Student's *t*-test.

c. Filamentation frequency was calculated based on the percentage of filamentous mini-colonies at 21 hours after incubation on V8 agar. The data are presented as the mean ± SD from three independent experiments. Two-tailed Student's *t*-test.

d. Bilateral *CQS2* overexpression cell fusion frequency compared to wild-type. The data are presented as the mean ± SD from three independent experiments.

e. The colony morphology, self-filamentation and sporulation phenotypes of JEC21α and the JEC21α-derived *CQS2* overexpression strains. Hyphae and chains of basidiospores were photographed after 14 days of growth on V8 medium. Images are representative of three independent experiments conducted with similar results. Scale bars, 1 mm (upper and middle panels), 20 μm (bottom panel).

f. 3×10^4 cells of JEC21α or JEC21α-derived *CQS2* overexpression strains were spotted onto V8 medium and the number of sporulated basidia were measured after 14 days of incubation at 25 °C in the dark. Red line indicates the mean value from three independent experiments.

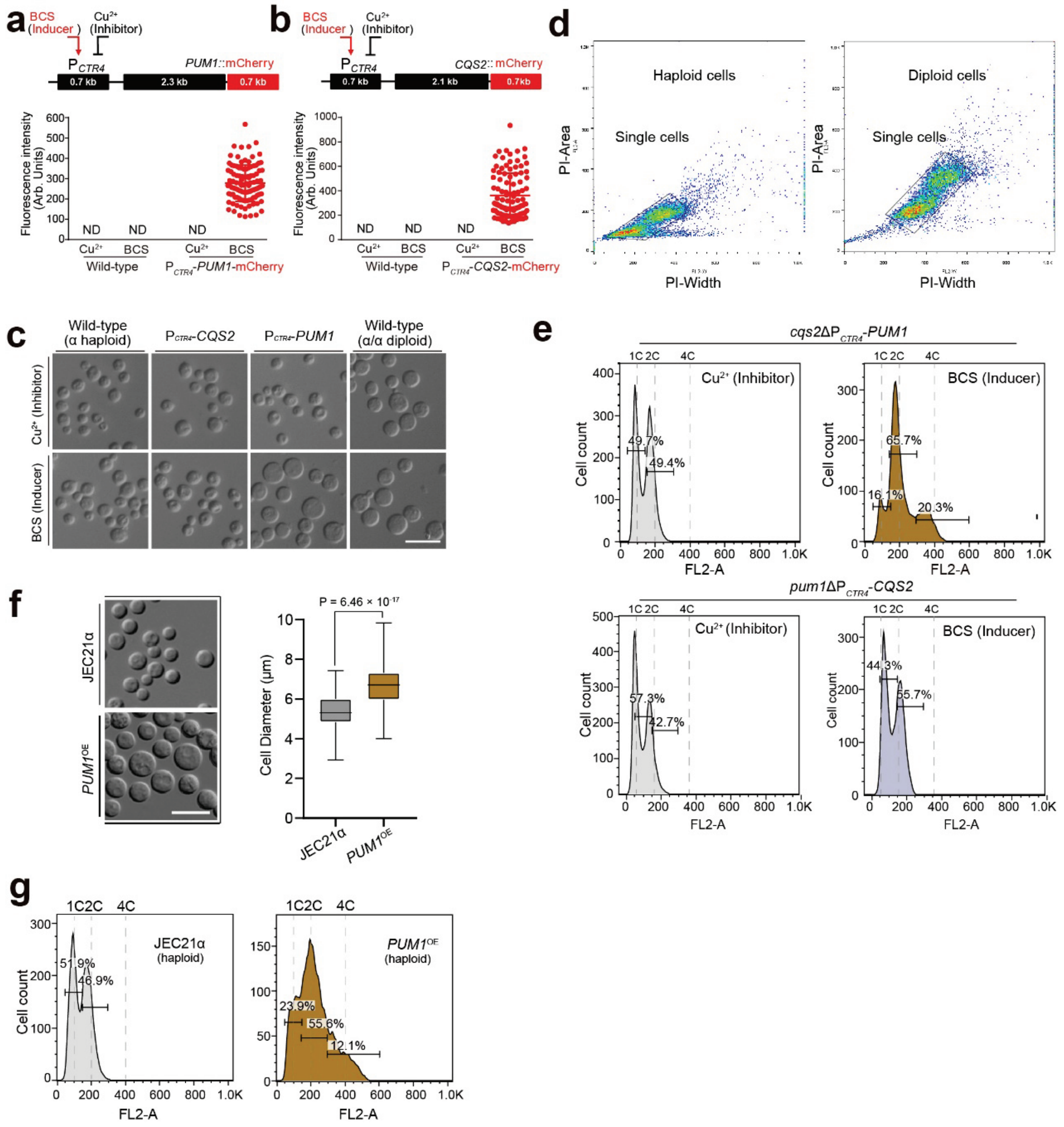

Supplementary Figure 5. Pum1 is a key target of Cqs2 during unisexual autodiploidization.

a. The expression of Pum1 in the P_{CTR4}-*PUM1*-mCherry strain under sex-suppressing condition (YPD liquid medium) in the presence of BCS or copper (n = 100 for each strain). Data are presented as the mean ± SD.

b. The expression of Cqs2 in the P_{CTR4}-*CQS2*-mCherry strain under sex-suppressing condition (YPD liquid medium) in the presence of BCS or copper (n = 100 for each strain). Data are presented as the mean ± SD.

c. Cellular morphology of different strains under sex-suppressing condition (YPD liquid medium) in the presence of BCS or copper. Images are representative of three independent experiments conducted with similar results. Scale bar, 10 μm.

d. Flow cytometry plots showing the gating strategy for single cells. Yeast cells were stained with Propidium Iodide (PI), and FL2W/FL2A was used for doublet discrimination.

e. FACS-based ploidy assessment of *cqs2*ΔP_{CTR4}-*PUM1* and *pum1*ΔP_{CTR4}-*CQS2* strain. Strains were cultured in YPD liquid medium for 3 days in the presence of copper (final concentration 25 μM) or BCS (final concentration 20 μM).

f. Cellular morphology (left) and cell size distribution (right) of JEC21α and JEC21α-derived *PUM1* overexpression strain cultured under sex-suppressing condition in the presence of 20 μM BCS. Images are representative of three independent experiments conducted with similar results. 100 cells of each strain were examined for their diameters. Boxplots show the median and the upper and lower central quartiles. The expected range of the data is indicated by whiskers. Scale bar, 10 μm. Two-tailed Student's t-test.

g. FACS-based ploidy assessment of JEC21α and JEC21α-derived *PUM1* overexpression strain. Strains were cultured in YPD liquid medium for 3 days in the presence of BCS (final concentration 20 μM) before FACS analysis.

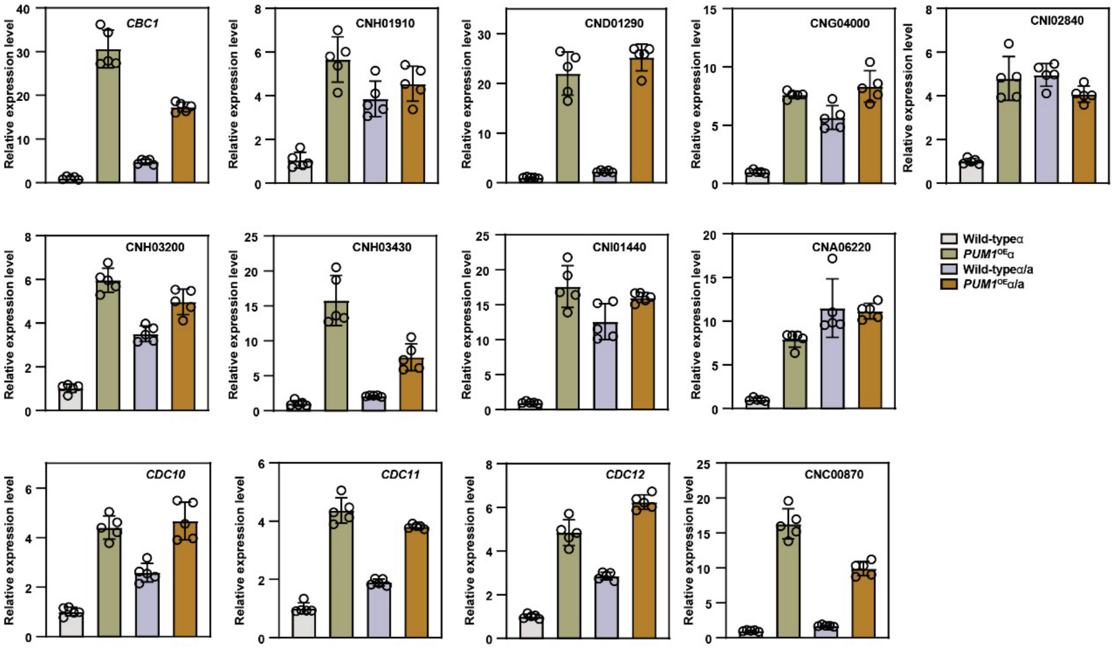

Supplementary Figure 6. **Ploidy determines the stimulatory effect of Pum1 on the expression of the cell cycle genes.** The mRNA levels of 13 cell cycle genes in different strains. The data are presented as the mean ± SD from five independent experiments.

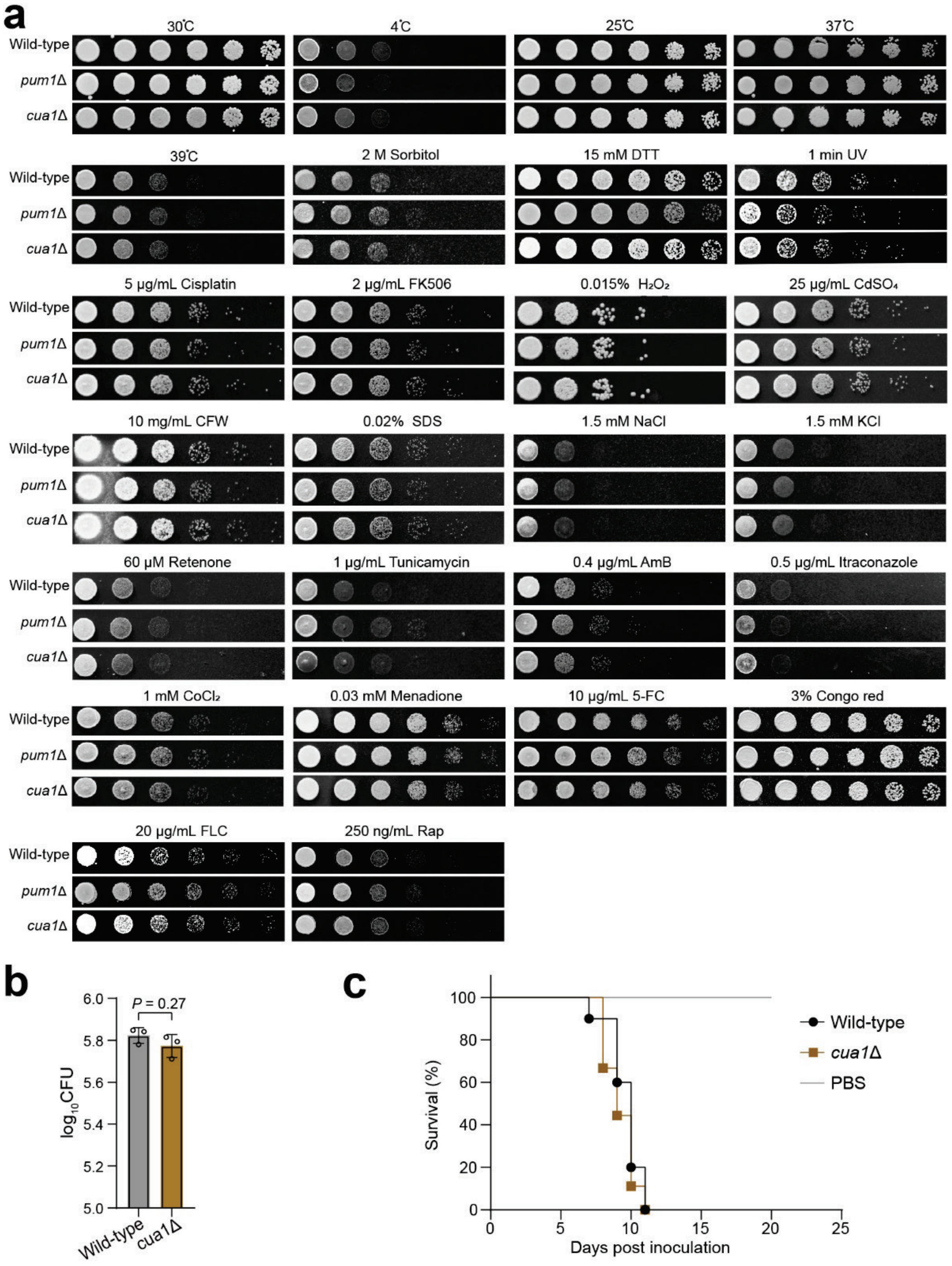

Supplementary Figure 7. **The absence of Pum1 or Cua1 does not cause remarkable changes in *C. deneoformans* under various stress conditions.**

a. Strains were grown overnight on YPD agar at 30°C, then 5-fold serially diluted and spotted onto media inducing different abiotic stress conditions. Osmotic stress (2 M sorbitol), cation/salt stress (1.5 M NaCl and 1.5 M KCl), cell wall stress (3% Congo red, 10 mg/mL CFW, and 0.02% SDS), ER and reducing stress (0.1 µg/mL tunicamycin and 15 mM DTT), oxidative stress (1.5 mM tert-butyl hydroperoxide and 30 µM menadione), heavy metal stress (25 µg/mL $CdSO_4$) antifungal stress (0.4 µg/mL Amphotericin B, 10 µg/mL 5-flucytosine, 20 µg/mL fluconazole, 250 ng/mL rapamycin, and 0.5 µg/mL itraconazole), genotoxic stress (1 min UV), hypoxic stress (25 µg/mL $CoCl_2$), temperature growth (4°C, 25°C, 30°C, 37°C and 39°C), and other stress (60

μM retenone).

b. Fungal burden in the brains of mice (n = 3) infected with wild-type or *cua1* mutant strains by intravenous tail vein injection was evaluated 7 days after infection. Data are presented as the mean ± SD. Two-tailed Student’s *t*-test.

c. Survival curves plotted against time upon challenge with wild-type or *cua1*Δ mutant in the *C. deneoformans* background by intravenous tail vein injection.
