# Supplementary material for: Regulatory basis for reproductive flexibility in a meningitis-causing fungal pathogen
Source: Nat Commun. 2022 Dec 24;13:7938. doi: 10.1038/s41467-022-35549-y (PMC9790007; doi:10.1038/s41467-022-35549-y)
Supplement: Supplementary file 3 — Description of Additional Supplementary Files [file 41467_2022_35549_MOESM3_ESM.pdf]

Description of Additional Supplementary Files:

Supplementary Data 1: List of C. deneoformans transcription factors predicted based on the DNA-binding domain database.

Supplementary Data 2: Heat map of eight phenotypic traits related to two sexual cycles in C. deneoformans TF mutants.

Supplementary Data 3: Genes differentially expressed in various mutant strains during unilateral mating.

Supplementary Data 4: Gene age and RNAseq data of genes in group 8.

Supplementary Data 5: Strain names and source information of Fmp1 homologs.

Supplementary Data 6: Genes differentially expressed in cpk1Δ and fmp1Δ mutant strains during unilateral mating.

Supplementary Data 7: Cqs2 direct regulon identified by the ChIP-seq.

Supplementary Data 8: Genes differentially expressed in CQS2 overexpression strain during unisexual reproduction.

Supplementary Data 9: Cell-cyle related genes that were upregulated by both Pum1 and Cqs2 based on RNA-seq analysis.

Supplementary Data 10: Strains used in this study.

Supplementary Data 11: Primers used in this study.
