# Supplementary material for: Regulatory basis for reproductive flexibility in a meningitis-causing fungal pathogen
Source: Nat Commun. 2022 Dec 24;13:7938. doi: 10.1038/s41467-022-35549-y (PMC9790007; doi:10.1038/s41467-022-35549-y)
Supplement: Supplementary file 15 — Reporting Summary [file 41467_2022_35549_MOESM15_ESM.pdf]

nature portfolio

Corresponding author(s): Linqi Wang

Last updated by author(s): Nov 25, 2022

Reporting Summary

Nature Portfolio wishes to improve the reproducibility of the work that we publish. This form provides structure for consistency and transparency in reporting. For further information on Nature Portfolio policies, see our Editorial Policies and the Editorial Policy Checklist.

Statistics

For all statistical analyses, confirm that the following items are present in the figure legend, table legend, main text, or Methods section.

n/a	Confirmed	
[ ]	[x]	The exact sample size (n) for each experimental group/condition, given as a discrete number and unit of measurement
[ ]	[x]	A statement on whether measurements were taken from distinct samples or whether the same sample was measured repeatedly
[ ]	[x]	The statistical test(s) used AND whether they are one- or two-sided<br>*Only common tests should be described solely by name; describe more complex techniques in the Methods section.*
[x]	[ ]	A description of all covariates tested
[x]	[ ]	A description of any assumptions or corrections, such as tests of normality and adjustment for multiple comparisons
[ ]	[x]	A full description of the statistical parameters including central tendency (e.g. means) or other basic estimates (e.g. regression coefficient) AND variation (e.g. standard deviation) or associated estimates of uncertainty (e.g. confidence intervals)
[ ]	[x]	For null hypothesis testing, the test statistic (e.g. F, t, r) with confidence intervals, effect sizes, degrees of freedom and P value noted<br>*Give P values as exact values whenever suitable.*
[x]	[ ]	For Bayesian analysis, information on the choice of priors and Markov chain Monte Carlo settings
[x]	[ ]	For hierarchical and complex designs, identification of the appropriate level for tests and full reporting of outcomes
[ ]	[x]	Estimates of effect sizes (e.g. Cohen's d, Pearson's r), indicating how they were calculated

Our web collection on statistics for biologists contains articles on many of the points above.

Software and code

Policy information about availability of computer code

Data collection	AxioCam MRm camera software Zen 2011, BD CellQuest™ Pro v6.0, Ultrahigh-resolution laser confocal microscopy (Nikon A1-N-SIM S)
Data analysis	GraphPad Prism 8.0, AxioCam MRm camera software Zen 2016, R statistical platform (version 3.4.2), FlowJo v10.0, Integrative Genomics Browser 9.0.0, PyMOL version 2.5.0, Bowtie2 (version 2.1.0), MACS2 (ver. 2.1.1), ChIPseeker (version 1.10.3), HOMER (4.9.1), Nikon NIS-Elements AR Ver5.40.00 (Nikon Corporation), Gephi software v0.9.3, Cytoscape software v3.8.0, MMseqs (2.0), AlphaFold2 version 2.1.1, FigTree v1.4.4, IQ-TREE v2.1.2, RAxML-ng v0.9, trimAl v1.4, MUSCLE v3.8, SAMtools (version 1.5), Stringtie v1.3.3, DEseq2 v1.16.1, FastQC v0.11.5, STAT_2.6.0c.

For manuscripts utilizing custom algorithms or software that are central to the research but not yet described in published literature, software must be made available to editors and reviewers. We strongly encourage code deposition in a community repository (e.g. GitHub). See the Nature Portfolio guidelines for submitting code & software for further information.

Data

Policy information about availability of data

All manuscripts must include a data availability statement. This statement should provide the following information, where applicable:

- Accession codes, unique identifiers, or web links for publicly available datasets
- A description of any restrictions on data availability
- For clinical datasets or third party data, please ensure that the statement adheres to our policy

All data needed to evaluate the conclusions in the paper are present in the paper or in Supplementary Information. The sequencing data has been deposited at the

Gene Expression Omnibus (GEO) with accession number GSE135566 (https://www.ncbi.nlm.nih.gov/geo/query/acc.cgi?acc=GSE135566).

Human research participants

Policy information about studies involving human research participants and Sex and Gender in Research.

Reporting on sex and gender	N/A
Population characteristics	N/A
Recruitment	N/A
Ethics oversight	N/A

Note that full information on the approval of the study protocol must also be provided in the manuscript.

Field-specific reporting

Please select the one below that is the best fit for your research. If you are not sure, read the appropriate sections before making your selection.

☒ Life sciences ☐ Behavioural & social sciences ☐ Ecological, evolutionary & environmental sciences

For a reference copy of the document with all sections, see nature.com/documents/nr-reporting-summary-flat.pdf

Life sciences study design

All studies must disclose on these points even when the disclosure is negative.

Sample size	No statistical methods were used to predetermine sample size. Sample sizes were estimated based on standards of this field and preliminary experiments. For in vitro experiment, 3 - 150 sample size was used for analysis; For in vivo experiment , 10 mice per group were used. The exact n for each experiment was described in corresponding figure legends. These sample sizes were sufficient to detect meaningful biological difference with good reproducibility.
Data exclusions	No data were excluded.
Replication	All results were repeated two,three, five or six independent times. Please refer to Figure Legend for the detailed explanation of each experiment. Similar data were obtained in independent experiments. All experimental findings reported in the paper were reliably reproduced during replicate experiments.<br>Two biological replicates were done for RNA-seq and ChIP-seq.<br>Five or six biological replicates were done for RT-PCR analysis.<br>There biological replicates were done for assessment of phenotypic traits.
Randomization	This is not relevant to the study because specific genotype backgrounds were used for all the experiments and not applying a treatment to a larger subset of species.
Blinding	This is not relevant to the study because there was not group allocation in the study.

Reporting for specific materials, systems and methods

We require information from authors about some types of materials, experimental systems and methods used in many studies. Here, indicate whether each material, system or method listed is relevant to your study. If you are not sure if a list item applies to your research, read the appropriate section before selecting a response.

Materials & experimental systems

n/a	Involved in the study	
☐	☒	Antibodies
☒	☐	Eukaryotic cell lines
☒	☐	Palaeontology and archaeology
☐	☒	Animals and other organisms
☒	☐	Clinical data
☒	☐	Dual use research of concern

Methods

n/a	Involved in the study	
☐	☒	ChIP-seq
☐	☒	Flow cytometry
☒	☐	MRI-based neuroimaging

Antibodies

Antibodies used	RFP-Trap® Magnetic Agarose, Nanobody, (rtma-20, ChromoTek) 1:200 dilution<br>Anti-FLAG® Magnetic Beads, clone M2 (M8823, Sigma-Aldrich) 1:200 dilution<br>FLAG tag antibody, clone M2 (F1804, Sigma-Aldrich) 1:1000 dilution<br>MBP-agarose beads, Nanobody, (mbta-20, Chromotek) 1:200 dilution<br>MBP tag monoclonal antibody, clone 4C6H4, (66003-1-Ig, Proteintech) 1:1000 dilution<br>HRP conjugated anti-mouse antibody, IgG+IgM (H+L), (BE0141, EasyBio) 1:10,000 dilution
Validation	MBP tag monoclonal antibody, clone 4C6H4, (66003-1-Ig, Proteintech) 1:1000 dilution<br>WB analysis of Recombinant protein using 66003-1-Ig<br>Recombinant protein were subjected to SDS PAGE followed by western blot with 66003-1-Ig (MBP tag antibody) at dilution of 1:4000 incubated at room temperature for 1.5 hours. Recommended dilution: Western Blot (WB) dilution: 1:1000-1:8000<br><br>MBP-agarose beads, Nanobody, (mbta-20, Chromotek) 1:200 dilution<br>Immunoprecipitation of maltose binding protein (MBP) from E. coli cell extract. The Western blot shows the very high effectivity of the MBP-Trap: No MBP is left in Flow-Through lane.I: Input, FT: Flow-Through, B: Bound. Immunoprecipitation of MBP-fusion proteins and their interacting factors with anti-MBP Nanobody conjugated to beads. Applications IP, CoIP, ChIP, RIP. Binding capacity 45 µg (0.625 nmol) of recombinant MBP-tagged protein (~74.2 kDa) per 25 µL bead slurry.<br><br>RFP-Trap® Magnetic Agarose, Nanobody, (rtma-20, ChromoTek) 1:200 dilution<br>RFP-Trap Magnetic Agarose for immunoprecipitation of RFP and mCherry fusion proteins.I: Input, FT: Flow-through, B: Bound. The ChromoTek RFP-Trap® Magnetic Agarose are affinity beads for IP of RFP-fusion proteins. It comprises a RFP Nanobody/ VHH coupled to magnetic agarose. Immunoprecipitation of RFP-fusion proteins and their interacting factors with anti-RFP Nanobody conjugated to magnetic agarose beads. 22.5 µg of recombinant RFP per 25 µL bead slurry.<br><br>Anti-FLAG® Magnetic Beads, clone M2 (M8823, SIGMA) 1:200 dilution<br>Anti-FLAG M2 Magnetic Beads are 4% agarose beads bound with the Anti-FLAG M2 (mouse monoclonal) antibody. The M2 antibody recognizes the FLAG sequence at the N-terminus, Met-N-terminus and C-terminus. This alows for detection and capture of fusion proteins containing a FLAG peptide sequence. Suitable for immunoprecipitation procedures. Supplied as a 50% suspension in 50% glycerol with 10mM sodium phosphate, 150mM sodium chloride, pH 7.4 and 0.02% (w/v) sodium azide (PBA/A). magnetic beads affinity isolated antibody. proteins (FLAG® Affinity Gels, FLAG® tag, 3x FLAG® tag, DYKDDDDK tag). superparamagnetic iron impregnated 4% agarose bead, with an average diameter of 50 µm.<br>≥0.6 mg/mL binding capacity<br><br>FLAG tag antibody, clone M2 (F1804, SIGMA) 1:1000 dilution<br>The ANTI-FLAG M2 mouse, affinity purified monoclonal antibody binds to fusion proteins containing a FLAG peptide sequence. The antibody recognizes the FLAG peptide sequence at the N-terminus, Met-N-terminus, C-terminus, and internal sites of the fusion protein. 1 mg/mL, clone M2, affinity isolated antibody, buffered aqueous solution (50% glycerol, 10 mM sodium phosphate, and 150 mM NaCl, pH 7.4). For highly sensitive and specific detection of FLAG fusion proteins by immunoblotting, immunoprecipitation (IP), immunohistochemisty, immunofluorescence and immunocyotchemistry. Optimized for single banded detection of FLAG fusion proteins in mammalian, plant, and bacterial expression systems. Western Blotting and EIA.

Animals and other research organisms

Policy information about studies involving animals; ARRIVE guidelines recommended for reporting animal research, and Sex and Gender in Research

Laboratory animals	C57BL/6 mice were purchased from Vital River (Beijing, China). All animal studies were performed with sex-(female) and age-matched (6-8 weeks) mice. The mice were routinely maintained in a pathogen-free animal facility at a temperature of 21°C, relative humidity of 50-70%. Mice were cared with an alternating 12 h light-dark cycle and unlimited food and water supply. Infected mice were sacrificed using carbon dioxide.
Wild animals	No wild animals were used in this study.
Reporting on sex	Sex was not considered in the study design. As there were no sex-based differences, female mice were used in the experiments due to increased ease of handing.
Field-collected samples	No field collected samples were used in this study.
Ethics oversight	All experiments involving mice were performed under the guidance of “the regulation of the Institute of Microbiology, Chinese Academy of Sciences of Research Ethics Committee.” The mouse models and procedures performed have been approved by the Institute of Microbiology, Chinese Academy of Sciences of Research Ethics Committee (Permit No. APIMCAS2021146).

Note that full information on the approval of the study protocol must also be provided in the manuscript.

ChIP-seq

Data deposition

☒ Confirm that both raw and final processed data have been deposited in a public database such as GEO.

☒ Confirm that you have deposited or provided access to graph files (e.g. BED files) for the called peaks.

Data access links<br>*May remain private before publication.*	https://www.ncbi.nlm.nih.gov/geo/query/acc.cgi?acc=GSE135566
Files in database submission	GSM4017302 FLAG IP_a<br>GSM4017303 FLAG IP input<br>GSM4017304 FLAG IP_b<br>GSM4017305 FLAG IP_b input<br>GSM4017306 mCherry IP_a<br>GSM4017307 mCherry IP_a input
Genome browser session<br>(e.g. UCSC)	https://www.ncbi.nlm.nih.gov/genome/61?genome_assembly_id=22728

Methodology

Replicates	Strains expressing Cqs2-Flag and Cqs2-mCherry with two biological repeats
Sequencing depth	Data was sequenced by paired-end and depth was 100x of genome.
Antibodies	RFP-Trap® Magnetic Agarose (rtma-20, ChromoTek)<br>Anti-FLAG® M2 Magnetic Beads (M8823, SIGMA).
Peak calling parameters	ChIP-Seq peaks were called using MACS2 (version 2.1.1.20160309) (Zhang et al., 2008) with default parameters
Data quality	sample Peak number<br>mCherry1 1203<br>mCherry2 1053<br>FLAG1 1614<br>FLAG2 1593
Software	Bowtie2 (version 2.1.0), MACS2 (ver. 2.1.1), ChIPseeker (version 1.10.3), HOMER (4.9.1).

Flow Cytometry

Plots

Confirm that:

☒ The axis labels state the marker and fluorochrome used (e.g. CD4-FITC).

☒ The axis scales are clearly visible. Include numbers along axes only for bottom left plot of group (a 'group' is an analysis of identical markers).

☒ All plots are contour plots with outliers or pseudocolor plots.

☒ A numerical value for number of cells or percentage (with statistics) is provided.

Methodology

Sample preparation	Yeast cells were stained with Propidium Iodide(PI) as described in Methods.
Instrument	BD FACSCalibur.
Software	Acquisition: BD CellQuest™ Pro v6.0, analysis: FlowJo v10.0.
Cell population abundance	Not applicable as no cells were sorted - they were only analysed.
Gating strategy	FL2W/FL2A was used for doublet discrimination. Haploid and diploid yeast cells strained with propidium iodide were used as haploid and diploid control, respectively.

☒ Tick this box to confirm that a figure exemplifying the gating strategy is provided in the Supplementary Information.
